# Geometrical Structures and Electronic Properties of Ga_6_ and Ga_5_X (X = B, C, N, O, F, Al, Si, P, S, Cl) Clusters

**DOI:** 10.3390/ma11040552

**Published:** 2018-04-04

**Authors:** Yanfei Hu, Guangfu Ji, Yachuan Yao, Jiaonan Yuan, Weisen Xu

**Affiliations:** 1 National Key Laboratory for Shock Wave and Detonation Physics Research, Institute of Fluid Physics, China Academy of Engineering Physics, Mianyang 621999, China; huyanfei1982@126.com (Y.H.); jackxws@live.cn (W.X.); 2 School of Physics and Electronic Engineering, Sichuan University of Science & Engineering, Zigong 643000, China; ty5288@126.com; 3 Institute of Atomic and Molecular Physics, Sichuan University, Chengdu 610065, China; jiaonanyuan@163.com

**Keywords:** density functional theory, CALYPSO, Multiwfn

## Abstract

Based on the unbiased CALYPSO (Crystal structure Analysis by Particle Swarm Optimization) structure searching method in combination with density functional theory (DFT), the geometrical structures and electronic properties are investigated theoretically for Ga_6_ and Ga_5_X (X = B, C, N, O, F, Al, Si, P, S, Cl) clusters. The PBE0 exchange-correlation functional and the 6-311G(d) basis set is carried out to determine global minima on potential energy surfaces. The relative stabilities of the clusters are examined by the binding energies and substitution reaction. Following the predictions of the Jellium model, the Ga_5_B cluster with the 18 valence electrons is the most stable structure. At last, with the obtained lowest energy structures, some physical properties such as electrons transfer, molecular orbitals, and total and partial densities of states are discussed, respectively.

## 1. Introduction

Due to the unusual structure and bonding characteristics, gallium has attracted a significant amount of attention for its extensive application in the thin-layer growth and the thin-film deposition in the deposition of multilayer structures [1,2,3]. In the last decade, many theoretical and experimental studies of gallium clusters have been reported [4,5,6,7,8,9,10,11]. For example, based on the density functional theory (DFT), the lowest-energy geometrical and electronic structures of Ga_n_ clusters containing up to 26 atoms have been calculated with generalized gradient approximation for the exchange-correlation potential [4]. By a simulated annealing technique with ab initio molecular dynamics (AIMD) method, Gong and Tosatti determined the lowest energy structures of small Ga_n_ (*n* ≤ 8) clusters [6]. The atomic structure, structural transformation, and reactivity of Ga_13_ clusters were investigated by the first principles’ pseudopotential calculations [7]. With a similar approach, Jones obtained the most stable structures of Ga_n_ cluster up to *n* = 10 [9]. Based on eight different density functional theory and hybrid DFT/Hartree–Fock methods, the structures, dissociation energies and electron affinities of the anionic and neutral gallium clusters were calculated by Zhao et al. [10]. For the experimental research, photoelectron spectra of small anionic gallium clusters were presented in the size range of *n* = 1–15 [12]. Results show that a change in the pattern of the spectra near *n* = 6 can be predicted as a transition from planar to compact three-dimensional structures. A study of the electronic spectrum of the gallium dimer was presented in the range of 33,600–36,800 cm^−1^ [13]. In addition, Himmel et al. have characterized isolated Ga_2_ dimers in an argon matrix with the aid of resonance Raman and UV/Vis spectroscopy [14]. Then, Balducci et al. characterized the dissociation energies of Ga_2_ cluster, which derived from the Knudsen cell-mass spectrometric [15].

In order to modify or tune physical and chemical properties of clusters, doping, which is the intentional incorporation of impurities into materials, is a widely used strategy. When X (X = B, C, N, O, F, Al, Si, P, S, Cl) atom was doped into Ga_6_ clusters, the introduction of a doped atom in Ga_6_ clusters can change undoubtedly its geometrical structures and electronic properties, and further affect its chemical and physical properties significantly. A number of gallium clusters doped with a single X (X = N, O, F, Al, Si, P) atom have been observed and studied [16,17,18,19,20,21,22,23,24,25,26]. Based on the density functional theory (DFT), Costales et al. have studied the structures of Ga_n_N_n_ (*n* = 1–6) clusters [16,17]. Combined with molecular dynamics and simulated annealing techniques, Song calculated the structures of Ga_n_N_n_ (*n* = 3, 5, 6) clusters using a full-potential linear-muffintin-orbital method [18,19,20]. The Ga_n−1_Al^+^ clusters were generated by pulsed laser ablation of a liquid aluminum/gallium alloy [21]. By an all-electron linear combination of atomic orbital approach, low-lying isomers of Ga_n_Al (*n* = 1–15) clusters were reported [22]. The ground state structures, stability and electronic properties were systemically investigated for neutral, cationic and anionic Ga_n_O_n_ (*n* = 4–7) clusters [23]. The geometrical structures, electronic states and energies of Ga_n_P_m_ (*n* = 1–7; *n* = 1, 2) clusters were investigated using the density functional theory [24]. The equilibrium geometries and electronic states of Ga_3_Sn, GaSn_3_ and their ions were investigated using the complete active space self consistent field (CASSCF) levels and DFT [25]. Using ab initio quantum chemistry, the feasibility of laser cooling GaF was performed. The X^1^∑^+^, ^3^∏ and ^1^∏ states of GaF was calculated using the multireference configuration interaction (MRCI) level of theory [26]. However, a systematic study on X-doped gallium (X = B, C, S, Cl) clusters has not been reported, and some problems on the Ga_5_X (X = B, C, S, Cl) clusters also have not been solved either. For example, how does the structure of the gallium clusters change with the X atom doped, and how can the pure gallium cluster properties be changed after doping a single X atom?

In cluster physics, the Jellium model predicts that small clusters with a certain valence electronic configuration (8, 18, 20, 34, 40, 58…), which were known as magic numbers, exhibit increased stability compared with their neighboring configuration. However, some works have shown that the number of electrons not corresponding to the magic numbers also produces extra stability [27,28]. The nitrogen-doped (Ga_n_N, *n* = 1–9) clusters were investigated at the PBE/DNP level of theory. It is found that Ga_n_N (*n* = 3, 7, 15) clusters were particularly stable despite of the fact that these clusters do not conform to the predictions of the Jellium model because they have 14, 26 and 50 valence electrons. In addition, Rebere et al. reported that Ag_13_^−^, Ag_14_, and Ag_15_^+^ clusters with 14 valence electrons were resistant to reactivity with O due to their large highest occupied-lowest unoccupied molecular orbital (HOMO-LUMO) energy gaps, despite not having fully filled electronic shell configurations. Inspired by the above studies, when there are X-doped (X = B, C, N, O, F, Al, Si, P, S, Cl) gallium clusters, do the stable X-doped structures possess the magic number? Hence, it is of interest to carry on more detailed studies on the magic numbers.

With this purpose in mind, we systematically studied the geometrical structures and electronic properties of Ga_6_ and Ga_5_X (X = B, C, N, O, F, Al, Si, P, S, Cl, Ga) clusters based on a particle swarm optimization algorithm combined with density function theory. For all of these clusters, relative stability was measured via analysis of binding energies and substitution reaction. Based on the HOMO-LUMO gaps and chemical hardness, the stable structures were determined for Ga_5_X (X = B, C, N, O, F, Al, Si, P, S, Cl, Ga) clusters. At last, the electron transfer, molecular orbitals, and density of states are also computed and discussed.

## 2. Computational Detail

It is well known that the number of possible geometric configurations of clusters increases exponentially with the size of the clusters, it will be more complex for binary clusters. Thus, it is necessary for us to search for an effective method in structure prediction. In the present paper, we performed through the intelligent methodology [29,30], as implemented in the CALYPSO (Crystal structure Analysis by Particle Swarm Optimization) code [30]. The algorithm can predict stable structures depending only on the chemical composition. It has been successful in correctly predicting structures for various systems [31,32,33,34]. The optimized structures obtained from particle swarm optimization (PSO) are taken as the initial structures for further simulation by the DFT method. Within the CALYPSO structure search, each generation contains 20 structures, 70% of which are generated by a particle swarm optimization algorithm. The others will be generated randomly. We followed 50 generations for each cluster to achieve convergence of the potential energy surface sampling. Then, 1000 structures of variable structures can be obtained for every Ga_5_X (X= B, C, N, O, F, Al, Si, P, S, Cl) and Ga_6_ clusters. The further geometry optimizations are performed with the PBE0 functional using the 6-311G(d) basis set, as implemented in the Gaussian09 package [35]. PBE0 has been confirmed to be suitable for describing the energy difference of isomers of X-doped gallium clusters [36]. In the process of calculation, the effect of the spin multiplicity is performed in the geometric optimization procedure. Harmonic vibrational frequencies are calculated to ensure that the obtained optimized structures are real minima with no imaginary frequencies. 

The accuracy of the present DFT method was assessed by benchmark calculations for available experimental and theoretical results. For Ga_2_, the calculated dissociation energy and vibrational frequency are 1.34 eV and 214 cm^−1^, respectively, in excellent agreement with experimental values 1.50 eV and 180 cm^−1^ [14,37]. To our knowledge, there is no bond length in the experiment for the Ga_2_ dimer. The present Ga–Ga bond length (2.45 Å) is broadly consistent with the previous theoretical studies [4,6,7,9]. For GaAl and GaSi, we obtained a bond length (2.456 Å, 2.43 Å) that fits well with the theoretical values of 2.585 Å and 2.45 Å by the GGA using B3LYP function, respectively [23,38]. In addition, for Ga_6_ cluster, the calculated vertical electron affinity (VEA) (2.12 eV) and vertical ionization potential (VIP) (6.69 eV) are close to the experimental values (2.60 eV) and theoretical vertical ionization potential (6.52 eV), respectively [12]. In short, PBE0/6-311G(d) level is able to describe the structural and electronic properties of Ga_6_ and Ga_5_X (X = B, C, N, O, F, Al, Si, P, S, Cl) clusters in a satisfactory manner. 

## 3. Results and Discussion

### 3.1. Lowest Energy Structures and Growth Pattern

**Figure 1:**
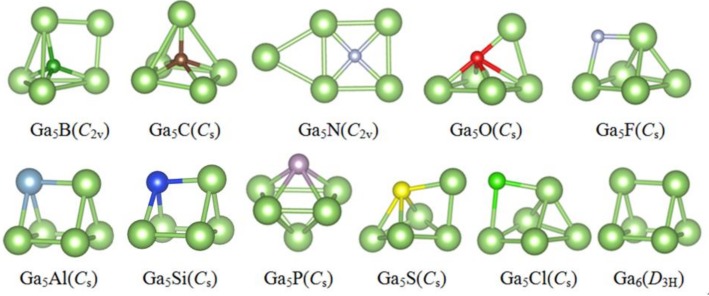
Optimized geometries of the lowest energy Ga_5_X (X = B, C, N, O, F, Al, Si, P, S, Cl, Ga) clusters.

The lowest energy structures of Ga_6_ and Ga_5_X clusters determined from CALYPSO and DFT calculation are displayed in Figure 1. The corresponding electronic states, symmetries and harmonic vibrational frequencies are tabulated in Table 1, and the atomic coordinates of each cluster are provided in the Supporting Information (SI) for the lowest energy structures.

As for the Ga_6_ cluster, the ^3^A_1_′ state of the trigonal prism (*D*_3h_) is almost degenerate with the ^1^A_1_ state of the similar trigonal prism, and the latter is computed to be 0.003 eV lower in energy, which is a certainly insignificant amount. Our result agrees with Drebov and Jones et al.’s results [9,39]. However, Gong and Tosatti applied an ab initio molecular-dynamics method and showed the most stable structure to be a distorted prism structure with *C*_2v_ point symmetry [6]. 

For a Ga_5_Al cluster, using the B3LYP-DFT calculations, Guo et al. predicted that the ground state is *C*_s_ structure and analogous to Ga_6_ structure, which is identical to our results [23]. Simultaneously, the calculated the vertical ionization potential (VIP) of 6.70 eV and the vertical electron affinity (VEA) of 2.13 eV in our work agree with previous DFT estimates (6.44 and 2.11 eV) calculated by Guo et al. [23].

Li et al. [25] calculated geometrics of Ga_n_P cluster and predicted that the ground state is the trigonal bipyramidal state (*C*_s_). From Figure 1, we can find that our result is identical to Li et al. In addition, our calculation predicted the VEA of the ground state (1.68 eV) agree with Li’s results (1.93 eV).

For the Ga_5_N cluster, in Li’s calculation, the ground state structure with *C*_2v_ point symmetry is a planar structure. In our prediction, the result is similar to Li and Song’s results [20,40]. Moreover, the calculated intensity vibration and lowest frequency (631 and 27 cm^−1^) are close to the Li et al.’s results (672.3 and 32 cm^−1^) [40].

Although the ground state structures of the Ga_5_X (X = B, C, S, Cl) clusters have not been reported, the above structures are obtained and listed in Table 1. Comparing X-doped (X = B, C, N, O, F, Si, P, S, and Cl) clusters, we can find that the doped atom simply replaces one Ga atom of pure Ga_6_ cluster, leading to a reduction of the molecular symmetry for the lowest stable configurations of Ga_5_B(^1^A_1_), Ga_5_C(^2^A″), Ga_5_O(^2^A′), Ga_5_F(^1^A′), Ga_5_Al(^3^A″), Ga_5_Si(^2^A′), Ga_5_S(^2^A′) and Ga_5_Cl(^1^A′). On the contrary, completely new structures are formed for Ga_5_N(^1^A_1_) and Ga_5_P(^1^A′) clusters. In conclusion, except for Ga_5_N and Ga_5_P clusters, the dopants of lighter elements (B, C, O, F) greatly influence the structure of the pure gallium clusters; however, those of heavier elements (Al, Si, S, Cl) generally generate a minor effect.

### 3.2. Relative Stability

The relative stability of clusters can be compared with the computing the binding energy (BE). Binding energies are calculated by the enthalpy change for the following reaction:

5Ga + X→Ga_5_X. (1)

In addition, the stability can also be measured by the enthalpy of substitution reaction (∆H_subst_), which can be expressed as:

Ga_6_ + X→Ga_5_X + Ga. (2)

**Figure 2:**
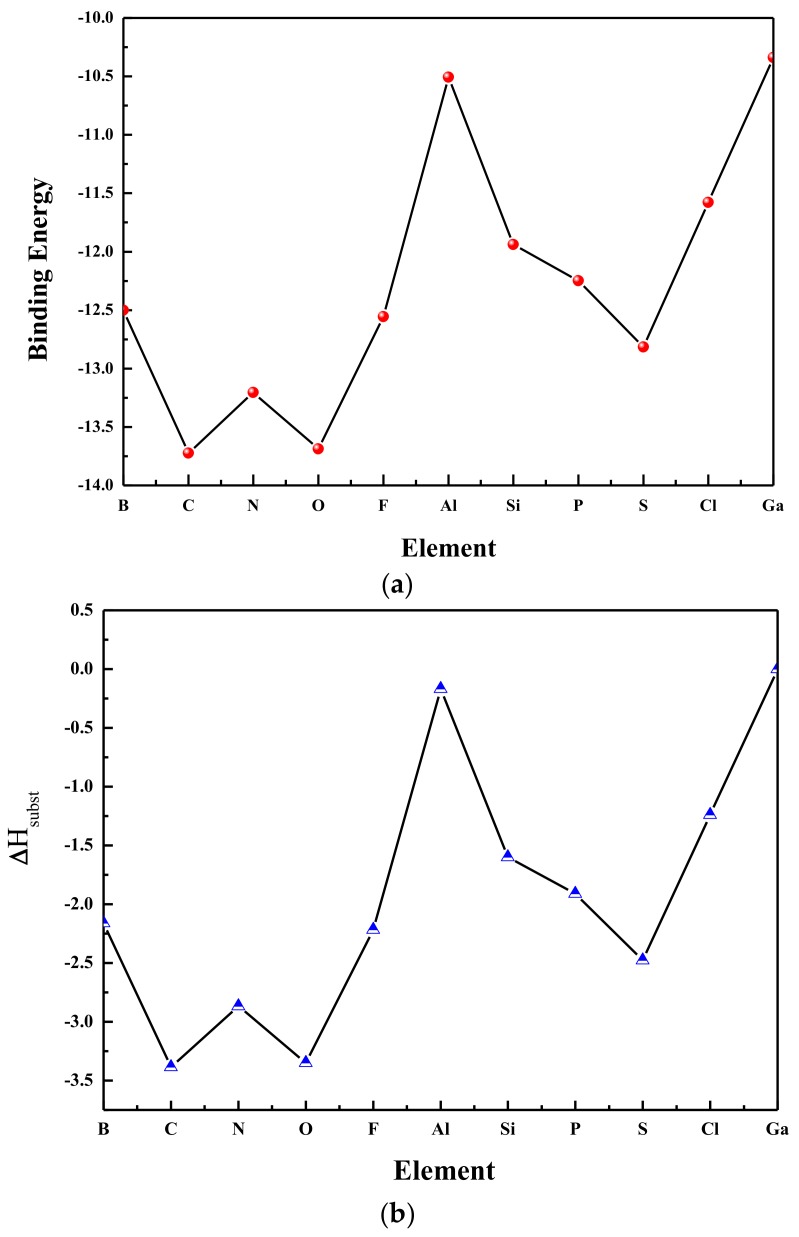
(**a**) Binding energies and (**b**) ∆H_subst_ of substituted gallium clusters (Ga_5_X) (eV).

In Figure 2, the values of the BE and ∆H_subst_, corrected for zero point vibrational energy, are shown with the cluster size increasing. 

As seen in Figure 2, the Ga_6_ cluster with 18 valence electrons possesses the maximum value of the binding energy. The isoelectronic Ga_5_Al and Ga_5_B clusters are also possessed of considerably larger binding energies, indicating greater overall stability. The Ga_5_Al cluster has larger binding energy than that of the Ga_5_B cluster. Moreover, this result can be obtained for all isoelectronic X-doped systems. In general, the tendency of ∆H_subst_ is similar to that of the binding energies, and the Ga_5_C cluster leads to the largest stabilizing effect (∆H_subst_ = −3.38 eV). Theoretically, the dopants of B and Al should arouse a minor influence on the stability of the doped clusters because they are equivalent to the valence electron of the Ga atom. In fact, we find that the Al atom actually leads to an increase in stability (0.16 eV) while the B atom leads to an even greater increase in stability (2.16 eV). Doping with O and S elements leads to a 21 valence electrons system and would not be expected to lead to a significant increase in stability. However, it is clear from Figure 2 that X-doped (X = O, S) clusters increase the stability of the cluster. Generally speaking, based on the BE and ∆H_subst_, the stability of host Ga_6_ clusters will indeed be enhanced by the dopants of X atoms. 

### 3.3. The HOMO-LUMO Gaps

In cluster science, the HOMO-LUMO gaps (*E*_gap_) can be usually used for estimating the chemical stability of the clusters. Larger values of the gaps indicate stronger chemical stability. In Table 1, we list the calculated energy gaps of the mentioned clusters. It is interesting to notice that the Ga_5_B, Ga_5_N, Ga_5_F, Ga_5_P and Ga_5_Cl clusters with an even number of electrons (18, 20, 22) have higher stabilities than other Ga_5_C, Ga_5_O, Ga_5_Si and Ga_5_S clusters with an odd number of electrons (19, 21). For Ga_5_B, Ga_5_N, Ga_5_F, Ga_5_P and Ga_5_Cl clusters, all paired electrons form a closed-shell electronic structure caused the larger energy gap of 1.87–2.88 eV. However, the Ga_5_C, Ga_5_O, Ga_5_Si and Ga_5_S clusters show a small energy gap due to the LUMO occupied by a single electron. In addition, the local maxima of *E*_gap_ are found at Ga_5_B, suggesting that the Ga_5_B cluster possesses enhanced relative stability, and the clusters with 18 valence electrons are expected to be especially stable by the Jellium model. 

Chemical hardness, proposed by Pearson [41,42], can also be viewed as a parameter to measure the relative stability of the clusters. The chemical hardness (*η*) can be expressed as 

*η* = VIP − VEA. (3)

VIP and VEA are the vertical ionization potential and vertical electron affinity, respectively. From Table 1, one can see that the tendency and the extremum (Ga_5_B) of *η* completely accord with the above analysis based on the HOMO-LUMO gaps. 

### 3.4. Charge Transfer in the Ga_5_X Cluster

To probe into the internal charge transfer of these X-doped gallium clusters, we extended the charge populations on Ga and X atoms inferred from the Hirshfeld, Mulliken, natural population analysis (NPA) and Bader approaches based on the Multiwfn program (Tian Lu and Feiwu Chen, Beijing, China) [43]. As is shown in Table 2, there is less positive charge on Ga with less negative charge on X, expect for Ga_5_Al clusters. The results show that the values for the X (X = B, C, N, O, F, Si, P, S, Cl) atoms are almost negative. This means that the charge transfers from the Ga host to X (X = B, C, N, O, F, Si, P, S, Cl) atoms in the corresponding clusters because of the higher electronegativity of the X (X = B, C, N, O, F, Si, P, S, Cl) atom than that of the Ga atom (Ga:1.81 eV, B:2.04 eV, C:2.55 eV, N:3.04 eV, O:3.44 eV, F:3.98 eV, Si:1.90 eV, P:2.19 eV, S:2.58 eV, Cl:3.16 eV) [44]. However, the electronegativity of the Ga (1.81 eV) is more than that of the Al (1.61 eV) [44]. One can see that the charge transfers from Al atoms to Ga frames in Ga_5_Al clusters. This fact has also been observed in a number of gallium-containing clusters in earlier reports [31]. 

### 3.5. The Molecular Orbitals

**Figure 3:**
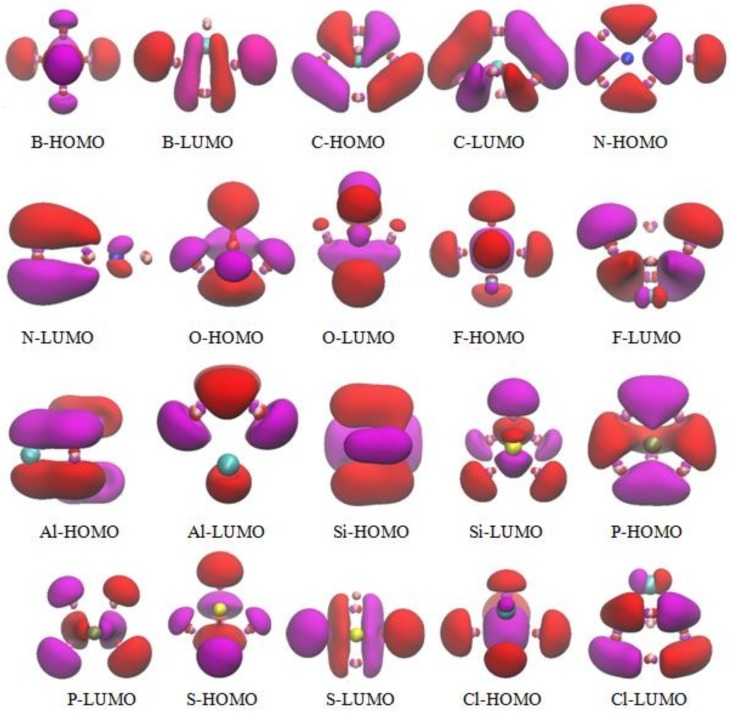
The molecular orbitals for the lowest energy structure of Ga_5_X (X = B, C, N, O, F, Al, Si, P, S, Cl) clusters.

In Figure 3, an examination of the molecular orbitals (MOs) is presented for understanding the shell model of the most stable Ga_5_X (X = B, C, N, O, F, Al, Si, P, S, and Cl) clusters. In an effort to obtain the specific orbital composition of every MO, the corresponding orbital compositions are also plotted in Figure S1 (see SI). 

From the Figure 3, it can be seen that the HOMO and LOMO are localized around the Ga atoms. For Ga_5_X (X = B, C, O) clusters, the HOMO involves the *s*, *p*_x_, *p*_y_ and *p*_z_ from all of Ga atoms, the *s*, *p_z_* of B atoms, the *p_z_* of C atoms; the contribution of *p*_y_ is very small from oxygen atoms. The LUMO of Ga_5_X (X = B, C) cluster is composed of (*s*, *p*_y_, *p*_z_), (*p*_x_, *p*_y_, *p*_z_) of Ga and *p*_y_ of B atoms; the effect of *p_z_* of C is very small. Moreover, the LUMO involves Ga-*s*, *p*_x_, *p*_y_ and *p*_z_ atomic orbitals as well as *p*_x_ and *p*_y_ from oxygen atom in Ga_5_O clusters. For Ga_5_F and Ga_5_Cl clusters, the HOMO mainly comes from the (*s*, *p*_z_) and (*s*, *p*_x_, *p*_z_) orbitals of Ga atoms along with small mixture of *p*_x_ and *p*_y_ orbitals in X (X = F, Cl) atoms. The LUMO is almost identical in Ga_5_F and Ga_5_Cl clusters. It results from the identical orbital. Gallium atoms involve *s*, *p*_x_, *p*_y_, and *p*_z_ component orbitals; F and Cl atoms possess the same *p*_z_ atomic orbitals. The HOMO of the Ga_5_Al and Ga_5_Si clusters is very similar. However, the HOMO includes the *p*_x_, *p*_y_, *p*_z_ states of the Ga atoms and the *s*, *p*_x_, *p*_y_ states of the Al atom in the Ga_5_Al cluster, whereas the HOMO mostly involves the *p*_x_ of gallium and silicon atoms as well as a small mixture of *s* orbitals of the Ga and Si atoms in the Ga_5_Si cluster. For Ga_5_X (X = N, P, S) clusters, the HOMO mainly comes from the same *p_y_*, and *p_z_* orbitals of Ga atoms in Ga_5_N and Ga_5_P clusters. In LUMO, all of the atoms are composed of the *p_x_*, *p_z_*, and *p_z_* atomic orbitals in Ga_5_N, Ga_5_P, and Ga_5_S clusters, respectively. The contribution of N, P, and S atoms are almost zero in HOMO and LUMO. These molecular orbitals indicate the presence of *sp* hybridization of the X atom and Ga atoms.

### 3.6. The Density of States

**Figure 4:**
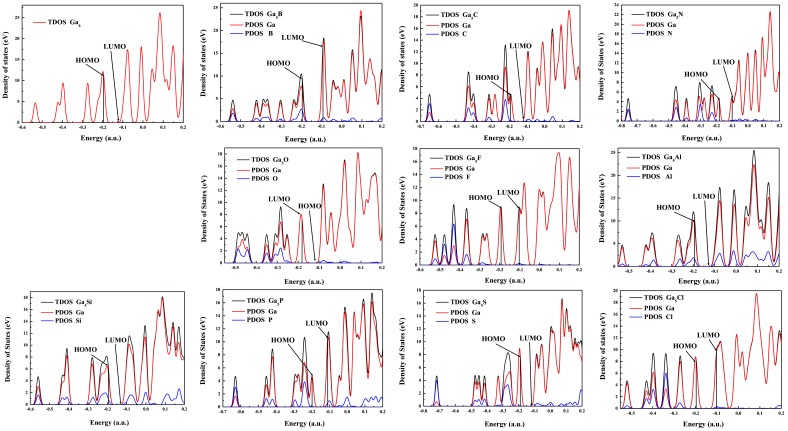
Calculated total densities of states (TDOS) and partial densities of states (PDOS) of Ga_5_X (X = B, C, N, O, F, Al, Si, P, S, Cl) clusters.

To understand the nature of the chemical bonding of these clusters, the density of states (DOS) of Ga_5_X (X = B, C, N, O, F, Al, Si, P, S, and Cl) and Ca_6_ clusters are shown in Figure 4. It is clear that there is finite electronic density, mainly coming from Ga atoms, at the Fermi level for all clusters, which means that they exhibit metallic behavior. At the Fermi level, the total density of states (TDOS) of Ga_5_X clusters is lower than that of the Ga_6_ cluster except for Ga_5_N and Ga_5_Si clusters. The fact indicates that the impurity atoms reduce the metallicity of the Ga_5_X (X = B, C, O, F, Al, P, S, and Cl) clusters. The contribution of the impurity atoms primarily centralizes in the valence bands for Ga_5_X (X = B, C, N, O, F, S, and Cl) clusters, and they are comparative in the valence bands and conduction band for Ga_5_X (X = Al, Si, and P) clusters. In addition, the HOMO energy levels shift to higher energy levels when X (X = C, N, O, F, and S) are doped gallium clusters, whereas they shift to lower energy levels when other atoms are doped gallium clusters.

## 4. Conclusions

In the present study, we have performed a global minimum search for the ground state structures of Ga_6_ and Ga_5_X (X = B, C, N, O, F, Al, Si, P, S, Cl) clusters by using the CALYPSO method in combination with DFT geometry optimization. The optimized geometries reveal that the dopant atoms (B, C, O, F, Al, Si, S, and Cl) simply replace one Ga atom of pure Ga_6_, leading to a reduction of the molecular symmetry. However, completely new structures are formed for Ga_5_N and Ga_5_P. Trends of the binding energies and substitution reaction showed that the dopant atoms can lead to increased stability relative to Ga_6_ clusters. Based on the HOMO-LUMO gaps and chemical harnesses, the Ga_5_B clusters with 18 valence electrons exhibit superior stability and can be viewed as the magic number clusters. In addition, analysis of molecular orbitals and DOS show that the HOMO and LOMO are localized around the Ga atoms, and the contribution of X atoms is almost zero. These molecular orbitals indicate the presence of *sp* hybridization of the X atom and Ga atoms. At last, the electrons transfer from the Ga frames to X (X = B, C, N, O, F, Si, P, S, Cl) atoms. On the contrary, the direction of charge transfer reverses and the amount of charges transfer from Al atoms to Ga atoms in Ga_5_Al clusters. 

## Figures and Tables

**Table 1:** State, symmetry, vertical ionization potential (VIP) (eV), vertical electron affinity (VEA) (eV), *η* (eV)*,* E_gaps_ (eV), and harmonic vibrational frequencies (cm^−1^) of Ga_5_X (X = B, C, N, O, F, Al, Si, P, S, Cl and Ga) clusters.

Isomer	State	Sym	VIP	VEA	*η*	E_gaps_	Frequencies	
							Lowest Frequency	Intensity Vibration
Ga_5_B	^1^A_1_	*C*_2v_	6.76	1.16	5.60	2.879	36(a_2_)	543(a_1_)
Ga_5_C	^2^A″	*C*_s_	6.67	1.95	4.72	1.934	15(a″)	547(a′)
Ga_5_N	^1^A_1_	*C*_2v_	6.44	1.56	4.88	2.241	27(b_1_)	631(a_1_)
Ga_5_O	^2^A′	*C*_s_	6.38	1.79	4.59	1.812	46(a′)	449(a′)
Ga_5_F	^1^A′	*C*_s_	6.64	1.49	5.15	2.360	60(a′)	424(a′)
Ga_5_Al	^3^A″	*C*_s_	6.70	2.13	4.57	1.874	28(a″)	170(a′)
Ga_5_Si	^2^A′	*C*_s_	6.79	2.16	4.63	1.836	18(a″)	207(a″)
Ga_5_P	^1^A′	*C*_s_	6.88	1.68	5.2	2.387	45(a″)	336(a′)
Ga_5_S	^2^A′	*C*_s_	6.65	2.05	4.6	1.837	30(a″)	289(a′)
Ga_5_Cl	^1^A′	*C*_s_	6.74	1.45	5.29	2.536	44(a′)	274(a′)
Ga_6_	^3^A_1_′	*D*_3h_	6.70	2.12	4.58	1.889	29(a_1_′)	115(e′)

**Table 2:** Theoretical atomic charges on X atoms (X = B, C, N, O, F, Al, Si, P, S, Cl).

Methods	B	C	N	O	F	Al	Si	P	S	Cl
Hirshfeld	−0.399	−0.436	−0.405	−0.366	−0.274	0.001	−0.175	−0.224	−0.248	−0.177
Mulliken	−0.861	−1.350	−1.150	−0.822	−0.471	0.254	0.023	−0.061	−0.156	−0.262
NPA	−2.865	−1.395	−2.179	−0.789	−0.755	0.189	−0.332	−0.921	−0.503	−0.485
Bader	−1.282	−1.816	−1.673	−1.343	−0.773	0.350	−0.371	−0.857	−0.974	−0.591

## Supplementary Materials

The following are available online at http://www.mdpi.com/1996-1944/11/4/552/s1, Figure S1: The orbital composition of HOMO and LUMO for Ga_5_X (X = B, C, N, O, F, Al, Si, P, S, Cl) clusters.
