# Supplementary material for: Geometrical Structures and Electronic Properties of Ga6 and Ga5X (X = B, C, N, O, F, Al, Si, P, S, Cl) Clusters
Source: Materials (Basel). 2018 Apr 4;11(4):552. doi: 10.3390/ma11040552 (PMC5951436; doi:10.3390/ma11040552)
Supplement: Supplementary file 1 [file materials-11-00552-s001.pdf]

Supporting Information

Geometrical Structures and Electronic Properties of Ga_6 and Ga_5X (X = B, C, N, O, F, Al, Si, P, S, Cl) Clusters

Yanfei Hu [1,2], Guangfu Ji [1,*], Yachuan Yao [2], Jiaonan Yuan [3] and Weisen Xu [1]

1 National Key Laboratory for Shock Wave and Detonation Physics Research, Institute of Fluid Physics, China Academy of Engineering Physics, Mianyang 621999, China; huyanfei1982@126.com (Y.H.); jackxws@live.cn (W.X.)
2 School of Physics and Electronic Engineering, Sichuan University of Science & Engineering, Zigong 643000, China; ty5288@126.com
3 Institute of Atomic and Molecular Physics, Sichuan University, Chengdu 610065, China; jiaonanyuan@163.com
* Correspondence: cyfjkf@caep.ac.cn; Tel.: +86-813-550-5296

Cartesian coordinates for the lowest-energy structures of Ga_6 and Ga_5X (X=B, C, N, O, F, Al, Si, P, S, Cl) clusters.

Ga_6

Ga	0.00000000	1.54089100	1.27868600
Ga	-1.33445100	-0.77044500	1.27868600
Ga	0.00000000	1.54089100	-1.27868600
Ga	-1.33445100	-0.77044500	-1.27868600
Ga	1.33445100	-0.77044600	1.27868600
Ga	1.33445100	-0.77044600	-1.27868600

Ga_5B

Ga	0.00000000	1.81807000	0.58270900
Ga	1.72106800	0.00000000	-1.73878800
Ga	0.00000000	0.00000000	2.38339000
Ga	0.00000000	-1.81807000	0.58270900
Ga	-1.72106800	0.00000000	-1.73878800
B	0.00000000	0.00000000	-0.44163100

Ga_5C

Ga	-1.47979900	-1.49821900	0.00000000
Ga	2.13092600	0.41950100	0.00000000
Ga	0.04027100	-0.53711300	1.97829700
Ga	-0.73946300	2.10972700	0.00000000
Ga	0.04027100	-0.53711300	-1.97829700
C	0.04027100	0.22329100	0.00000000

Ga_5C

Ga	0.00000000	1.41834500	-2.15198600
Ga	0.00000000	-1.41834500	-2.15198600
Ga	0.00000000	1.40263400	0.64615200

Ga	0.00000000	0.00000000	3.16739100
Ga	0.00000000	-1.40263400	0.64615200
N	0.00000000	0.00000000	-0.68962400

Ga_5O

Ga	-1.43053400	1.66629300	0.00000000
Ga	0.31445000	-0.38014700	1.82450900
Ga	-1.52453000	-1.03929100	0.00000000
Ga	2.24501600	-0.07530000	0.00000000
Ga	0.31445000	-0.38014700	-1.82450900
O	0.31445000	0.80829600	0.00000000

Ga_5F

Ga	0.04973400	-0.43776400	1.89657900
Ga	0.04973400	-0.43776400	-1.89657900
Ga	2.03758000	0.08007800	0.00000000
Ga	-1.65771400	-1.10246700	0.00000000
Ga	0.04973400	1.58018000	0.00000000
F	-1.82234200	1.09442400	0.00000000

Ga_5Al

Ga	-0.60482900	-1.14736900	1.33231100
Ga	1.70574700	-1.13350600	0.00000000
Ga	-0.60482900	1.41306900	-1.32617100
Ga	-0.60482900	-1.14736900	-1.33231100
Ga	-0.60482900	1.41306900	1.32617100
Al	1.70159200	1.43579200	0.00000000

Ga_5Si

Ga	0.58008100	-1.09678800	1.39246100
Ga	0.58008100	1.41596300	1.42178600
Ga	0.58008100	-1.09678800	-1.39246100
Ga	0.58008100	1.41596300	-1.42178600
Ga	-1.65924100	-1.17500200	0.00000000
Si	-1.46382900	1.18829900	0.00000000

Ga_5P

Ga	-1.62383600	-0.50624400	0.00000000
Ga	0.20388600	1.50914100	1.24487400
Ga	0.20388600	-1.20288500	1.60539100
Ga	0.20388600	1.50914100	-1.24487400
Ga	0.20388600	-1.20288500	-1.60539100
P	1.67047000	-0.21962300	0.00000000

Ga_5S

Ga	0.20075400	-0.42633800	1.92064000
Ga	0.20075400	-2.26285800	0.00000000
Ga	-1.08658700	2.18629400	0.00000000
Ga	0.20075400	-0.42633800	-1.92064000
Ga	1.22871900	1.03004900	0.00000000
S	-1.44226600	-0.19531800	0.00000000

Ga_5Cl

Ga	0.12664400	1.58679200	0.00000000
Ga	0.12664400	-0.42103100	1.89187400
Ga	0.12664400	-0.42103100	-1.89187400
Ga	-1.32934700	-1.48192100	0.00000000
Ga	2.13998600	0.13886400	0.00000000
Cl	-2.17103900	1.09106800	0.00000000

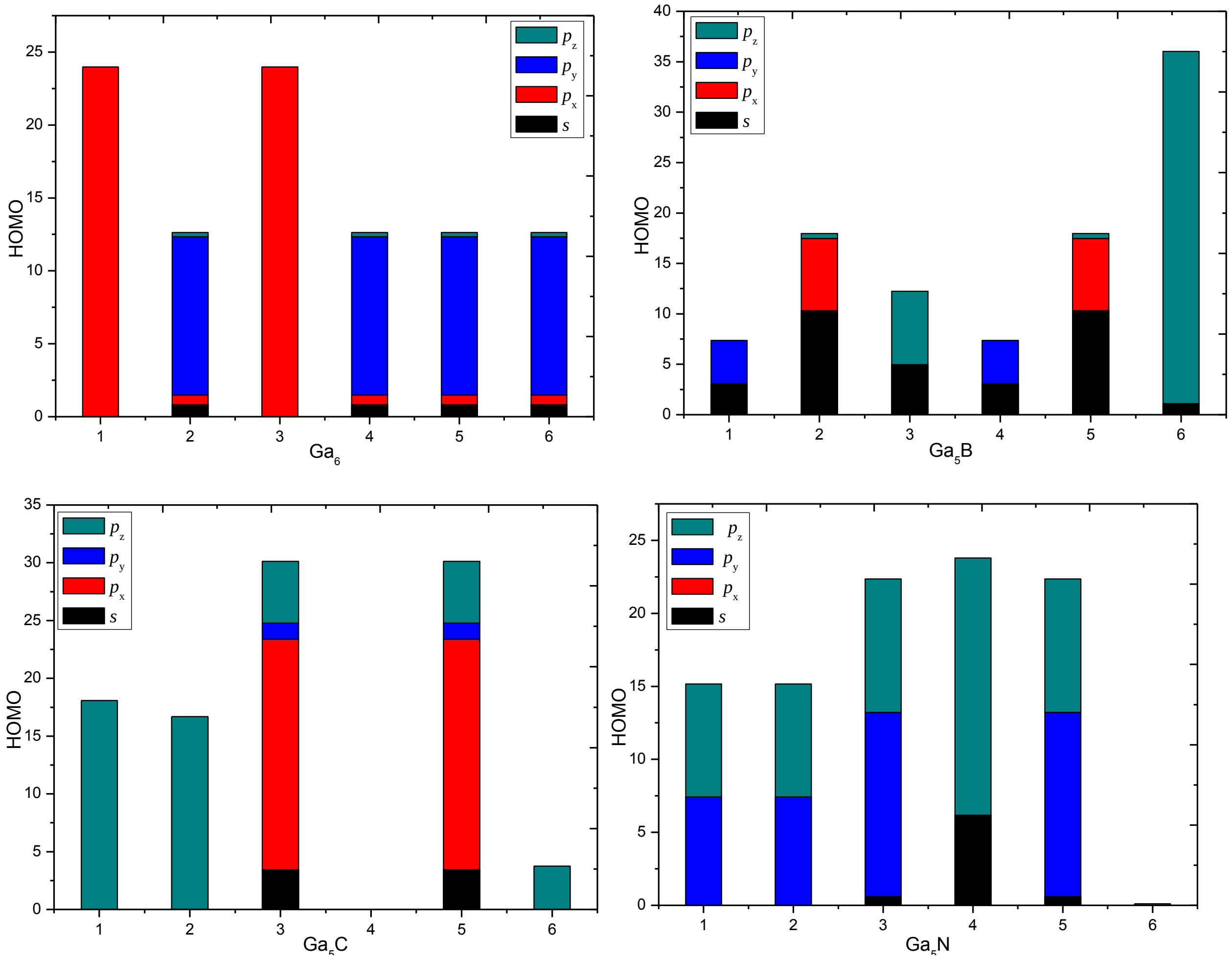

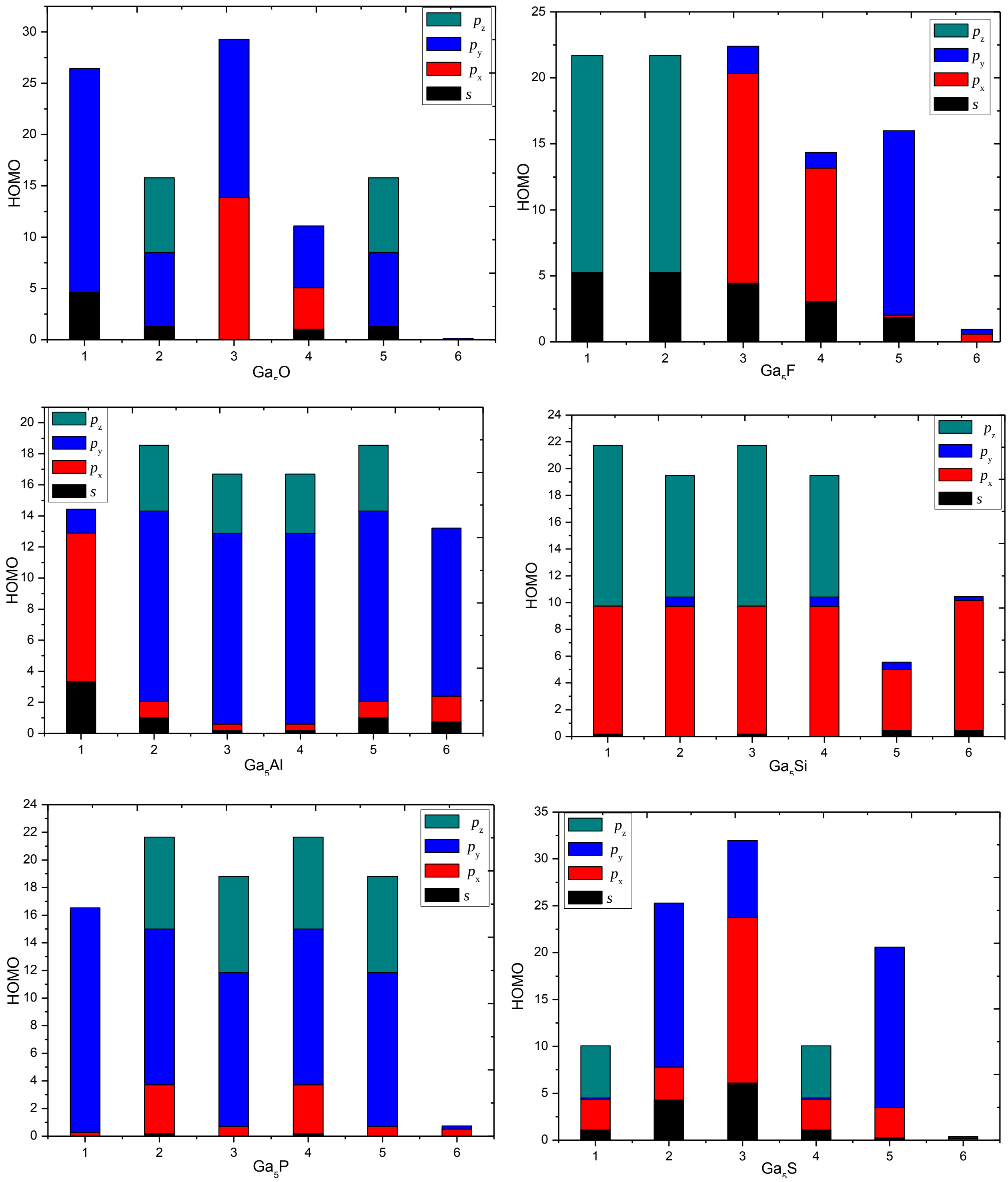
HOMO
pz
py
px
s
Ga5O
Ga5F
Ga5Al
Ga5Si
Ga5P
Ga5S

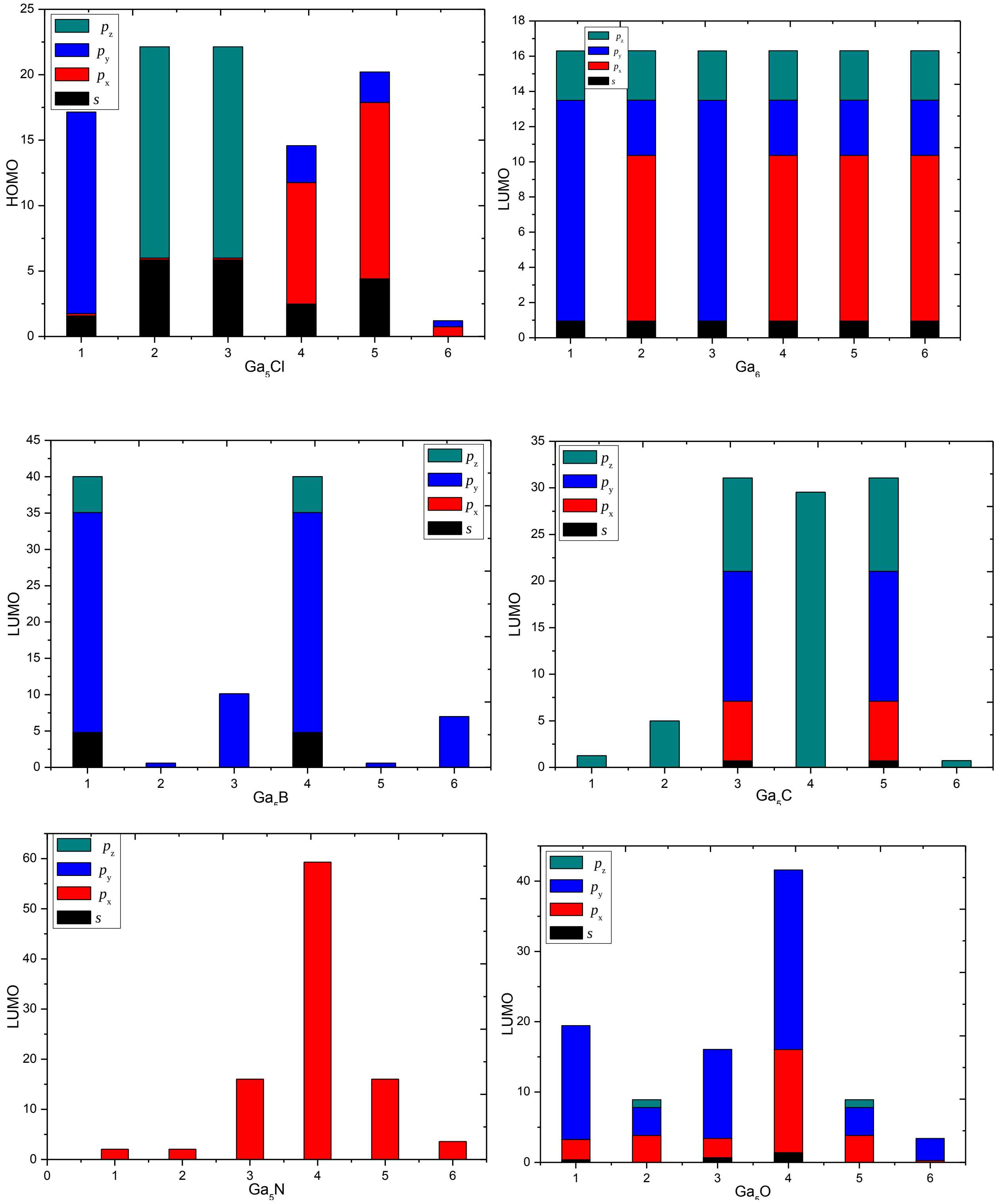
HOMO
Ga$_5$Cl
LUMO
Ga$_6$
Ga$_5$B
Ga$_5$C
Ga$_5$N
Ga$_5$O
p_z
p_y
p_x
s

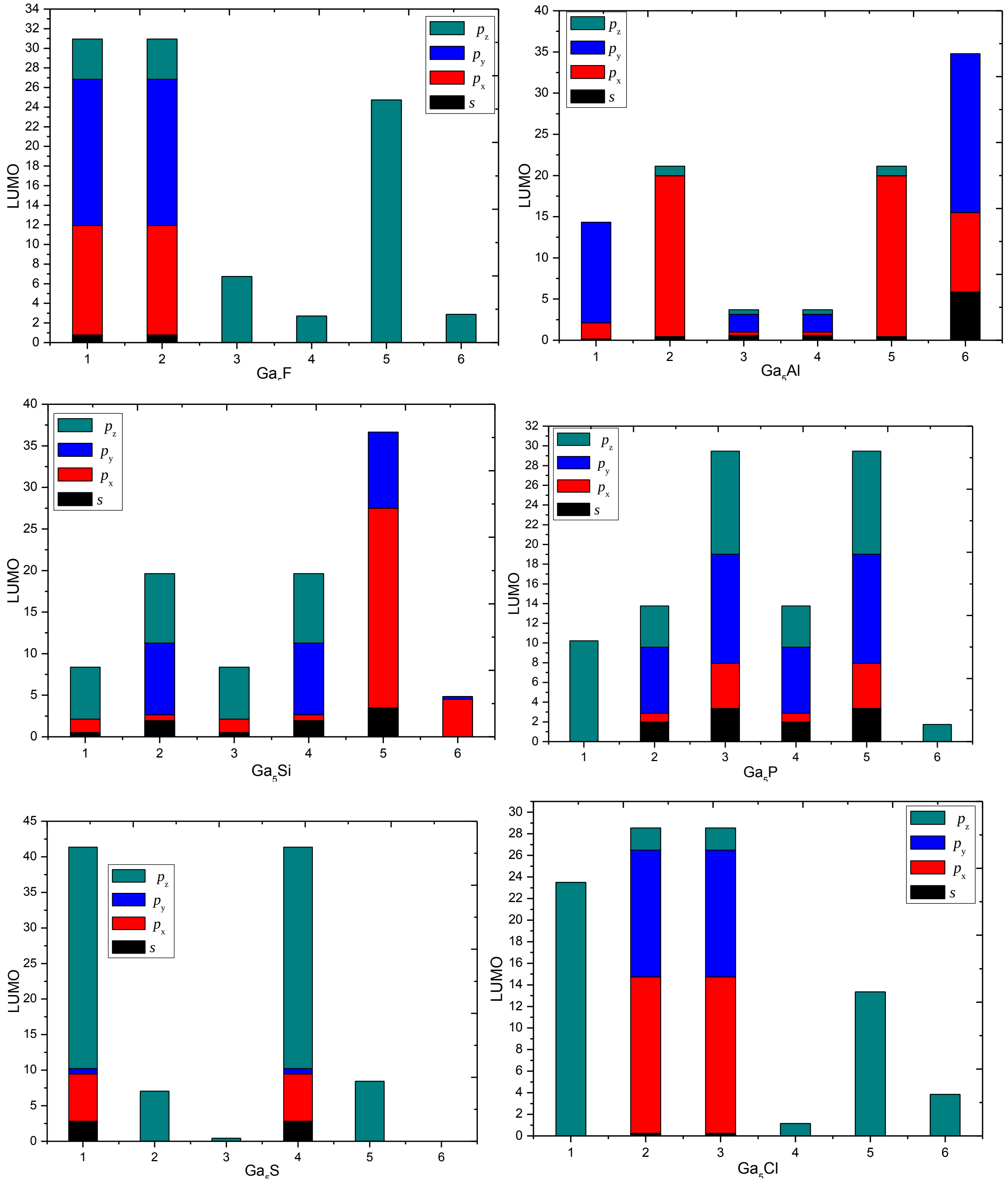

Figure S1. The orbital composition of HOMO and LUMO for Ga_5X (X=B, C, N, O, F, Al, Si, P, S, Cl) clusters.
